# Advances in CRISPR/Cas-Based Strategies on Zoonosis

**DOI:** 10.1155/2023/9098445

**Published:** 2023-08-03

**Authors:** Yi Zhang, Qifeng Peng, Renjun Zhang, Chao Li, Quangang Xu, Luming Xia, Youming Wang, Ping Liu, Hong Pan

**Affiliations:** ^1^Institute of Zoonosis, College of Public Health, Zunyi Medical University, Zunyi, China; ^2^Key Laboratory of Maternal and Child Health and Exposure Science of Guizhou Higher Education Institutes, Zunyi, China; ^3^Center for Animal Disease Control and Prevention of Guizhou Province, Guiyang, China; ^4^China Animal Health and Epidemiology Center, Qingdao, China; ^5^Center for Animal Disease Control and Prevention of Shanghai City, Shanghai, China

## Abstract

Clustered regularly interspaced short palindromic repeats (CRISPR)/CRISPR-associated (Cas) has emerged as the predominant technique for gene editing technique due to its high efficiency and low cost. In the area of zoonosis, CRISPR/Cas is also widely used in different research areas. This paper reviewed the principles of CRISPR/Cas technique and its applications in zoonosis. Moreover, we analyze the shortcomings and weaknesses that currently limit its use, highlight its direction for improvement, and foresee its application prospects in the prevention and treatment of zoonosis. For the purpose of preventing and controlling zoonosis, we need to develop diagnostic method with high sensitivity and specificity, highly protective vaccines, and also better understanding of the pathogenesis. Our review aimed to promote the application and improvement of CRISPR/Cas technique in the above-mentioned areas, and provide brief and comprehensive references for CRISPR/Cas-based research. Through reviewing the advances in CRISPR/Cas-based strategies on zoonosis, we believe that CRISPR/Cas technique will provide more powerful assistance for the prevention and control of zoonosis.

## 1. Introduction

New infectious diseases have emerged in recent years, including COVID-19, monkeypox, MERS, and, etc., most of the pathogens of which originated from animals. The global population is currently faced with over 200 zoonotic diseases, which not only pose significant threats to public health but also result in substantial economic losses [1]. There are also many unknown bacteria, viruses, and other microorganisms that can cause zoonotic diseases. Biological laboratories in several countries have discovered and isolated new pathogenic strains, which are highly transmissible and pathogenic and lack effective treatment, and are likely to cause bioterrorism if spread on a wide scale [2].

The prevention and control of zoonosis is not only of great significance for the development of animal husbandry, but also an important safeguard for global public health. In order to control zoonosis, we need to understand the pathogenesis and develop useful drugs or vaccines, and other tools with high efficiency. In particular, they are reliant on new techniques, of which gene editing technique is an important tool. Gene editing uses artificial means to alter DNA or RNA sequences for the purpose of changing biological phenotypes. This technique includes modification of gene sequences, introduction and integration of exogenous genes, and deletion of specific genes [3]. Gene editing is usually performed by constructing artificial endonucleases that cut DNA at predetermined genomic locations to generate double-strand breaks (DSBs). Mutations can subsequently occur during repair of DSBs by the intracellular DNA repair system, thus achieving targeted genome modification [4]. Currently, the commonly used endonucleases are mainly divided into four categories: clustered regularly interspaced short palindromic repeats (CRISPR)/CRISPR-associated (Cas), transcription activator-like effector nucleases (TALENs), zinc finger nucleases (ZFNs), and meganucleases (MNs) [5, 6].

MNs are enzymes that have highly specific DNA cleavage properties and long target recognition sequences [7]. They have proven to be invaluable tools for certain genome editing applications because of their monomeric nature, nonrepetitive sequence, and small size (180–440 residues, 18–40 kDa, coded in sequences of ∼1 kb), which also enables easy packaging and delivery in various forms, such as plasmids, mRNA, viral vectors, or proteins [6, 8, 9]. On the other hand, ZFNs are customizable and artificial nucleases that combine two functionally distinct domains. Their compact size (∼40 kDa) renders them well-suited for a wide range of delivery methods, including adeno-associated viral vectors to plasmids [8]. ZFNs offer the advantage of accommodating longer recognition sequences, which can be adjusted in proportion to the number of zinc fingers employed. Furthermore, despite originating from bacteria (FokI), ZFNs do not exhibit immunogenic epitopes [10], which plays a catalytic role in gene targeting modifications. TALENs as artificial restriction enzymes, are extensively utilized in various applications. They incorporate the catalytic module of FokI nucleases with the DNA-binding domain of TALEs, which are virulence proteins naturally secreted by plant pathogenic *Xanthomonas* bacteria [11]. TALENs have been widely used for genome editing, such as addition of N-terminal or C-terminal markers, base phosphorylation, base substitution, and point mutation. They have enabled targeted mutation of the genome in a variety of organisms, including rice, zebrafish, rats, mice, chicken, yeast, and viruses [12, 13].

Each platform presents notable limitations, but they also possess strengths that can render them suitable for specific application (Table 1). MNs are well-suited for scenarios that demand high precision, prioritizing accuracy over efficiency. Likewise, modified versions of ZFNs or TALENs incorporating mutated FokI domains can achieve highly efficient and specific modifications in target sequences. Nevertheless, these technologies are limited by complex retargeting procedures, which make them impractical as platform technologies for developing a wide range of genome editing applications. For example, while it is possible to use one of these techniques to develop a variant for a specific application, this approach may not be strategically favorable if the objective is to consistently target novel. Therefore, there is a need for more efficient and flexible genome editing technologies that can be easily retargeted to different sequences. Moreover, the challenges associated with protein engineering and cloning of ZFNs and TALENs have hindered widespread adoption of these tools among researchers [15].

Compared to other genetic editing techniques, CRISP/Cas system requires only a short RNA sequence that can recognize different target sequences, and the Cas endonuclease can perform cleavage without forming a dimer [14], so CRISPR/Cas offers remarkable versatility, as retargeting can be achieved through straightforward modification of the sgRNA. The wide range of Cas variants available, each with specific parameters and even additional functionality (such as transcriptional activators/repressors and base editors), further enhances the depth of the CRISPR/Cas platform. In addition, it has demonstrated compatibility with various delivery vehicles, including RNPs, viral vectors, and plasmids. Therefore, the CRISPR/Cas system has several advantages such as high throughput, high efficiency, and effectiveness in solving the problem of complex gene manipulation in microorganisms, plants, and animals, thereby showing huge application potential.

**Figure 1:**
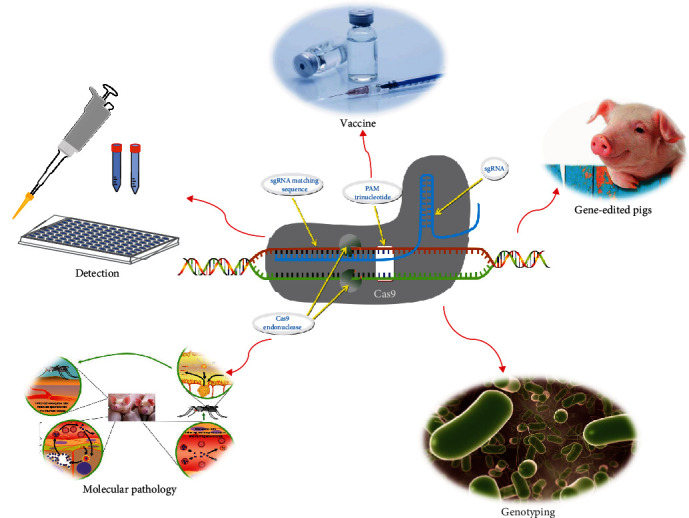
Applications of CRISPR/Cas system in zoonosis research applications of CRISPR/Cas system could be used in many areas of zoonosis research, mainly including the basic research, rapid diagnosis, vaccine development, and editing of disease-resistant genes.

Although other gene-editing techniques have been applied in some fields, they seem to not be as popular as CRISPR/Cas [16]. It has not been long since the CRISPR/Cas technique was developed. However, extensive investigation has been performed on its application in zoonosis and genetic modification of pathogenic microorganisms [17–19]. In this review, we examine studies focused on the applications of CRISPR/Cas in the field of zoonosis, including the basic research, rapid diagnosis, vaccine development, etc. (Figure 1). The aims of this review are to provide ideas and references for the subsequent research and application of this technique in the prevention and control of zoonosis.

## 2. Overviews of Crispr/Cas

### 2.1. The Classification of CRISPR/Cas System

So far, two classes of CRISPR/Cas systems have been developed, encompassing six types and multiple subtypes. The class 1 CRISPR/Cas systems consist of Types I, III, and IV, which employ an interference machinery comprising multiple Cas proteins. On the other hand, the Class 2 systems, comprising Types II, V, and VI, utilize a single Cas protein for interference (Table 2). Although a variety of CRISPR/Cas systems are available for targeted cleavage of target sequences, *Streptococcus pyogenes* (*S. pyogenes*) Cas9 (*Sp*Cas9) of Type II type is most widely used due to its straightforward requirement for 5′-NGG-3′ protospacer adjacent motif (PAM) sequence. CRISPR/Cas9 system comprises three components: Cas9 protein, trans-activating crRNA (tracrRNA), and CRISPR RNA (crRNA) [20].

### 2.2. The Principle and Process of CRISPR/Cas9

**Figure 2:**
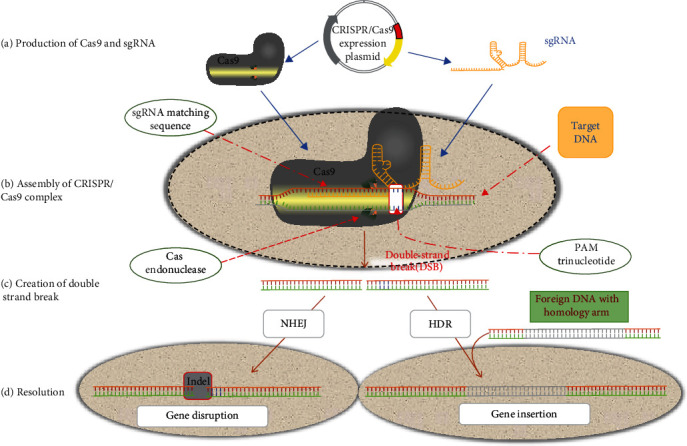
Mechanisms of CRISPR/Cas9 gene editing. (a) A construct that expresses a sgRNA segment and the Cas9 protein is introduced into the target cell. (b) The target DNA is bound by the sgRNA and Cas9 complex. (c) A double-strand break is introduced at the target site by the Cas9 nuclease. (d) The free ends of the DNA are subsequently repaired by the cell's DNA repair mechanisms, either through error-prone NHEJ, or with the addition of a homology template, through HDR.

Gene editing usually involves two steps: in the first step, guided by the sgRNA, the sgRNA and Cas9 seek out for the target across and create blunt-ended DSBs at approximately 3 bp upstream of the PAM site; in the second step, DNA is repaired in vivo employing either homology-directed repair (HDR) or error-prone nonhomologous end-joining (NHEJ) mechanisms [21] (Figure 2).

The initial step involves the sgRNA recognizing the target sequence through complementary pairing of sgRNA's 5′-crRNA with the base of the target gene. Subsequently, the sgRNA guides the Cas9 nuclease to the target sequence [22]. Cas9 possesses the DNA cleavage domains known as His–Asn–His (HNH) and RuvC, which are responsible for inducing DSBs in the DNA. These breaks occur primarily at the site located 3 bp upstream of PAM sequences (5ʹ-NAG for *Sp*Cas9 or 5ʹ NGG) within the target DNA [23]. The HNH domain cleaves the complementary strand of the crRNA, while the RuvC-like domain cleaves the opposite strand of the dsDNA [24]. Cas9 protein cuts DNA 3 bp upstream of the PAM sequence, creating a DSB. Subsequently, targeted DNA repairing mechanism usually involves NHEJ and HDR. In the absence of a donor DNA template, DSB is repaired through the NHEJ, and it may lead to random indels at the junction site, resulting in frameshift mutation or nonsense mutations, which is usually used for gene knockout [25]. HDR pathway achieves precise sequence through incorporating a donor DNA template that possesses sequence homology with a predicted DSB site, and it can realize that precise insertions or replacement in target genes can be made using exogenous reparative templates, which allows foreign DNA knock in or specific gene replacement [26].

## 3. Applications of CRISPR/Cas on Zoonosis

The enhanced comprehension of CRISPR/Cas biology has led to the broadened applications of this technique in the field of zoonosis. The CRISPR/Cas technique offers instruments that hold the potential to elucidate virulence-related gene, facilitate the development of fast and filed diagnostics, and improve the treatment and prevention of zoonosis.

### 3.1. Understanding the Virulence Gene

Understanding the pathogenesis of parasites, fungi, viruses, and bacteria is essential to the development of drugs, the formulation of vaccines, and the rational design of targeted therapies. For mechanism research of pathogens, gene editing and reverse genetic technique are essential tools. CRISPR/Cas-based gene editing has been extensively employed in various pathogens to investigate the roles of gene and protein in molecular pathogenesis.

#### 3.1.1. Virus

Animal viruses pose great threatens to animal husbandry, because of widely spreading and lack of therapeutic drugs. CRISPR/Cas system allows scientists to perform genetic manipulation on viral genes, such as targeted knockout, substitution, and codon modification, to obtain information on viral pathogenic genes, proteins, and their interactions with host proteins. Porcine reproductive and respiratory syndrome (PRRS) is a disease caused by PRRSV, which results in significant economic losses in the pig farming industry. PRRSV belongs to the *Arteriviridae* family, and possesses an enveloped, positive-sense single-stranded RNA genome [27]. In order to study the molecular pathogenesis of PRRSV, Na et al. [28] constructed a TALEN vector targeting the *NMHCII-A* gene and a CRISPR/Cas9 vectors expressed in the baculovirus expression system. They further characterized the basic properties of *NMHCII-A* gene-edited cell lines by examining the infectivity of PRRSV in the gene-edited cell lines combined with the gene function of *NMHCII-A*. Our study serves as a foundation for comprehending the invasion process of PRRSV and elucidating the dependent manner of interaction between PRRSV and *NMHCII-A* [29]. In another study, to examine the potential involvement of the endogenous protein p21 in PRRSV replication, Wen et al. [30] created a *p21* knockout MARC-145 cell line employing CRISPR/Cas9 system. Further studies using the MARC-145 cell line demonstrated that *p21* possesses an inhibitory effect on PRRSV replication, while PRRSV nsp11 protein facilitates *p21* degradation in an ubiquitin- and proteasome-independent manner [30]. Additionally, knockout of aminopeptidase N (APN) using CRISPR/Cas9 system showed that APN present on the surface of porcine intestinal villi is a key receptor for the infection of porcine transmissible gastroenteritis viruses (TGEV) [31]. Consistently, another study found that newborn piglets lacking APN protein are not susceptible to TGEV [32]. These studies indicated CRISPR/Cas system is a valuable instrument to understand the function of viral genes.

#### 3.1.2. Bacteria

CRISPR/Cas system can also be effectively employed for genetic manipulation on bacteria, including gene mutation, deletion, and replacement. Chen et al. [33] developed the pCasPA/pACRISPR genome editing method using phage *λ*-Red recombination and CRISPR/Cas9 system, which enables seamless and efficient genetic manipulation on *Pseudomonas aeruginosa*. In addition, they developed a base editing system called pnCasPA-BEC by fuzing cytidine deaminase APOBEC1 with endonuclease Cas9. This editing system enables point mutation and efficient gene inactivation in various *Pseudomonas* species including *P. aeruginosa* and *Pseudomonas putida*. The implementation of these genome editing methods is expected to greatly facilitate research in many areas, such as metabolic engineering, drug target discovery, and bacterial physiology. Synefiaridou and Veening [34] developed a replication plasmid carrying the CRISPR/Cas9 system and performed RNA-programmed genome editing by counterselection of the conditional pathogenic *Streptococcus pneumoniae*. After the introduction of a precise DSB, a HDR of the cellular DNAs was used to select successful transformants. The recombination of the DNA fragment occurred in the genome and target recognition of the endonuclease Cas9 was then eliminated. Notably, three modified strains with virulence deletion have been constructed using the above system [35]. This method has also been shown to be applicable to other *Gram-positive* bacteria, thus being potentially applied in a wider range of fields. Hong et al. [36] employed the CRISPR–Cpf1 to delete *Clostridium difficile* genes *fur*, *teM*, *and ermB1/2*, which are, respectively, involved in iron uptake, antibiotic resistance, and toxin production in bacteria. In addition to the above three genes, they deleted a very large gene locus of 49.2 kb, achieving efficient multiplex genome editing [36]. Therefore, this study facilitated further studies on the function of different loci in *C. difficile*.

RNA interference (RNAi) is limited to specific organisms, and the utilization of custom DNA-binding proteins can be challenging due to the high cost involved in their testing and designing. In contrast, CRISPR interference (CRISPRi) provides a cost-effective and straightforward approach that can be applied to regulate targeted gene expression in various microorganisms. CRISPRi system involves simultaneous expression of the catalytically inactive form of RNA-guided DNA endonuclease from the type II CRISPR system, known as dead Cas9 (dCas9), along with a small guide RNA specific to a target sequence. This coexpression leads to the formation of a DNA recognition complex that interferes with the transcription of the corresponding DNA sequence [37]. Choudharry et al. [38] showed that coexpression of the codon-optimized dCas9 of *S. pyogenes*, along with sequence-specific guide RNA, leads to the complete repression of individual or multiple targets in *mycobacterium tuberculosis*. Thus, CRISPRi provides a straightforward, fast, and cost-effective instrument for the selective control of gene expression in *mycobacteria*. Recently, growing evidence suggested the roles of CRISPR/Cas in bacterial virulence and resistance [39]. *Shewanella xiamenensis* is a newly identified zoonotic pathogen initially discovered in the coastal sea sediment in Xiamen, China [40]. Whole-genome shotgun sequencing revealed the presence of a CRISPR/Cas Type I–E system in *S. xiamenensis*, which encompasses a gene cluster comprising cas2, cas1, the cascade genes (casABCDE), and cas3, and the system may contribute to the fitness and pathogenesis of the bacteria [41]. Therefore, CRISPR/Cas is not only a tool for genetic editing, but also a target for virulence modification of bacteria.

#### 3.1.3. Parasite

As parasites have large genomes, editing their genomes requires high-throughput and high-efficiency gene-editing tools. Therefore, CRISPR/Cas is a good choice for parasitologists. By employing a transgenic line constitutively expressing Cas9, it was discovered that *EtGRA9* gene of *Eimeria tenella* encodes a secreted protein with varying cellular distribution throughout the parasite's life cycle. In addition, through systematic disruption of the ApiAp2 transcription factor gene family employing this method, it was uncovered that 23 of the 33 factors expressed by the parasite are crucial for its survival and development within the host [42]. Given that Cas9-induced DSBs fail to be repaired in *L. biflexa*, Fernandes et al. [43] employed a CRISPRi strategy to fulfill gene silencing by using catalytically dCas9. Coexpression of dCas9 and sgRNA targeting the coding strand of *β*-galactosidase gene in *L. biflexa* led to a complete gene silencing. This study represents the first application of the CRISPRi system to *leptospira* and *spirochetes* in general, thereby broadening the repertoire of tools accessible for *leptospira* research.

Genome-wide CRISPR screening is a powerful instrument that can be employed to identify genes under selective conditions. Nevertheless, the large scale of genome-wide libraries can impose limitation of application in experimental settings where cell numbers are constrained, such as single cell analysis or in vivo infections. To overcome this challenge, small scale CRISPR libraries targeting specific gene subsets can be utilized. Young et al. [44] developed a rapid method for generating custom guide RNA libraries utilizing arrayed single-stranded oligonucleotides. This approach enables reproducible pooled cloning of CRISPR/Cas9Cas9Cas9 libraries and was applied to create mutant pools of various sizes in the protozoan parasite *Toxoplasma gondi*. By using this approach, they conducted an in vivo genetic screen in the murine host, leading to the identification of several known and novel virulence factors that contribute to in vivo fitness [44]. CRISPR/Cas system enables gene editing in pathogenic microorganisms, thus facilitating the identification of virulence-associated proteins and amino acid sites, key proteins and regions responsible for pathogen-host interactions, and so forth.

### 3.2. Disease-Resistant Genetically Modified Animals

CRISPR/Cas system can be utilized to construct high-throughput mutation libraries, providing a effective instrument for facilitating investigation on resistance genes and breeding of disease-resistant animals. Luo et al. [45] employed CRISPR/Cas9 system combined with RNAi to generate swine fever-resistant transgenic pigs. In this experiment, shRNA was inserted into the pig *Rosa26* locus by CRISPR/Cas9 knock-in technique to degrade vRNA of classical swine fever virus (CSFV). The challenge experiments showed that the transgenic pigs infected with CSFV displayed significantly reduced virus replication, markedly decreased transmissibility of the virus, and significantly relieved clinical symptoms [45]. *RSAD2* gene has been considered as a potential knock-in candidate for the development of virus-resistant transgenic pigs, because of its broad antiviral activities against DNA and RNA viruses [46–48]. Xie et al. [49] specifically inserted porcine *RSAD2* gene into porcine ROSA26 locus (pROSA26) to generate transgenic pigs with reduced susceptibility to CSFV and pseudorabies virus (PRV). Whitworth et al. [50, 51] employed CRISPR/Cas9 system to generate *CD163* gene mutant pigs, which showed that the mutant pigs were resistant to PRRSV.

We could modify animal genes to reduce the susceptibility to pathogens using CRISPR/Cas system, and breeding disease-resistant animals is a good approach for disease prevention. Compared with the vaccine strategies, this approach could be a more economical and durable means of preventing infection of pathogenic microorganisms.

### 3.3. CRISPR/Cas-Based Diagnostics

Rapid and accurate diagnosis is the prerequisite and foundation for the prevention and control of zoonosis. The limit of CRISPR/Cas-based molecular detection could reach attomolar (aM) level, and its characteristics of accurate identification and field testing show great application potential [52]. Indeed, this technique has been investigated and applied in the detection and diagnosis of pathogenic microorganisms in recent years (Table 3).

#### 3.3.1. DETECTR

**Figure 3:**
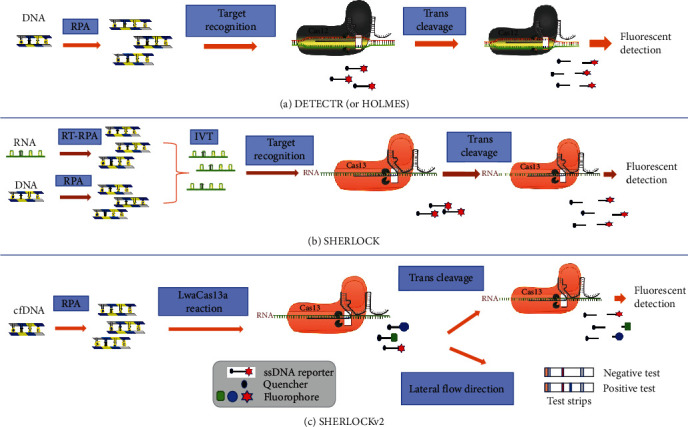
Schematics of CRISPR/Cas DETECTR diagnostic platform. CRISPR-based target nucleic acids detection involves multiple steps, which include the amplification of target nucleic acids using RPA, the generation of CRISPR/Cas activators or templates, Cas-based trans-cleavage of reporters, and signal detection. For target DNA, it can be directly amplified using DNA-dependent DNA polymerases. On the other hand, RNA is typically reverse transcribed into cDNA before undergoing amplification. (a) For Cas12-based DETECTR and HOLMES, the amplified products can be directly recognized and bound by Cas12 (including both Cas12a and Cas12b), triggering Cas12 trans-cleavage activities. (b) For Cas13-based SHERLOCK, the target is amplified by RPA with either RNA or DNA as the input (reverse RT–RPA or RPA, respectively). RPA products are detected in a reaction mixture containing T7 RNA polymerase, Cas13, a target-specific crRNA, and an RNA reporter that fluoresces when cleaved. Cas12 and Cas14 trans-cleave ssDNA probes, which can be dual labelled with a fluorescent unit at one end and a quencher unit at the other, while Cas13 has RNA collateral cleavage activities only and uses RNA probes in nucleic acid detection. (c) In the Cas13-based SHERLOCKv2 method, it is possible to achieve multiplex detection by using designed probes. Another option is to visualize reaction results on lateral flow strips utilizing chromogenic reaction. IVT, in vitro transcription; cfDNA, cell-free DNA.

Chen et al. [53] developed a method known as DNA endonuclease-targeted CRISPR transreporter (DETECTR) based on recombinase polymerase amplification (RPA) combined with Cas12a. The DETECTR allows direct detection of DNA amplicons and identification of viral DNA extracted from samples without additional transcription (Figure 3). An example of the application of CRISPR technology is the development of the DETECTR system for ASFV detection. DETECTR combines RPA reactions with CRISPR/Cas12a enzymes, specifically targeting conserved regions of the *p72* gene of ASFV. It was used to rapidly and specifically detect ASFV, and showed a minimum detection limit of eight copies. Importantly, no cross reactivity was observed with other main swine viruses, including JEV, PPV, PRV, PCV2, and CSFV [57]. In another study, by coupling with RPA and loop-mediated isothermal amplification assay, the DETECTR methods for detection of influenza A virus (IAV) and influenza B virus (IBV) were developed. *M* gene of IAV and HA gene of IBV was recognized by CRISPR/Cas12a system, and the limitation of the detection could reach as low as 1 *Pfu* per reaction without exhibiting cross-reactivity. For the reason of isothermal amplification, the whole process does not require additional analytic equipment [47]. In addition, Broughton et al. [58] reported a user-friendly, rapid (<40 min), and accurate method for detecting SARS-CoV-2 using CRISPR–Cas12-based lateral flow assay from respiratory swab RNA extracts. The result showed that CRISPR-based DETECTR assay offers a faster and visual alternative to the SARS-CoV-2 real-time RT–PCR assay. The DETECTR assay demonstrated a 95% positive predictive agreement and 100% negative predictive agreement.

#### 3.3.2. SHERLOCK

Combining CRISPR/Cas13a and RPA, the specific high-sensitivity enzymatic reporter unlocking (SHERLOCK) system was developed, which enables rapid detection of RNA and DNA viruses [52] (Figure 3). For example, the SHERLOCK system of CPV-2 was established, combining with RPA and T7 transcription. The method could detect 100 amol/L CPV-2 DNA within 30 min, and demonstrated high specificity [57]. In another example, the SHERLOCK system for white spot syndrome virus diagnostic was developed, based on RPA, synthetic biology approaches, and CRISPR detection, and the detection of limit could reach a single copy without sophisticated equipment [59]. Moreover, Lee et al. [60] established a diagnostic method for *Plasmodium falciparum* based on the SHERLOCK detection platform, which could distinguish among *P. falciparum*, *Plasmokjdium intercalans*, *Plasmodium ovale*, and *Plasmodium trisporus*. In addition, SHERLOCK platform has been employed for the detection of SARS-CoV-2. Typically, these methods involve a two-step process consisting of target amplification followed by CRISPR-based nucleic acid detection [53, 61]. Nevertheless, these approaches tend to be more complex, involving RNA extraction and multiple liquid-handling steps, which increase the risk of cross-contamination. To address this issue, Joung et al. [62] described a simple test for detection of SARS-CoV-2, named STOP (SHERLOCK testing in one pot). The sensitivity of this test is comparable to RT–qPCR assays. STOP is a streamlined assay that combines simplified extraction of viral RNA with isothermal amplification and CRISPR-mediated detection. This test can be performed at a single temperature in less than 1 hr and with minimal equipment.

SHERLOCK has shown high sensitivity and specificity in detecting target RNA. Nevertheless, it had limitations such as the inability to provide quantitation and reliance on fluorescence detection equipment for readout. To overcome these limitations and enhance the utility of the platform, Gootenberg et al. [54] further improved the SHERLOCK technology, which was named SHERLOCKv2. In this setting, the presence of the target is determined by visually inspecting the strips, which exhibit different intensities of staining. One of the key advantages of SHERLOCKv2 is that the entire reaction can be performed in a single step, allowing direct application of the biological sample to the test strip without the need for nucleic acid purification and isolation (Figure 3). To sum up, SHERLOCKv2 is a highly sensitive quantitative diagnostic platform that offers colorimetric detection and multiplex signal detection on lateral flow strips.

#### 3.3.3. HOLMES

Detection of DNA sequences using SHERLOCK requires in vitro transcription of DNA into RNA prior to the testing, thereby limiting its application. To overcome this deficiency, Li et al. [14] developed the 1 hr low-cost multipurpose highly efficient system (HOLMES) by utilizing quenched fluorescent single-stranded DNA (ssDNA) reporters as a probe, which can be used for rapid detection of target DNA and RNA with the sensitivity up to aM level. Further studies showed this method can be utilized for detecting DNA virus such as PRV, and RNA virus such as, JEV [63] (Figure 3). Moreover, HOLMES demonstrates high specificity and the ability to discriminate between virus strains. For instance, the Bartha-K61 vaccine strain, the cmz variant strain, and the PRV Ra classical strain were easily discriminated by the *gE*46 site. Similarly, the live-attenuated vaccine strain SA14-14-2 and the JEV NJ2008 strain were well-differentiated by the site *E*138 using HOLMES [63]. While both HOLMES and SHERLOCK can be employed for detecting DNA and RNA targets, HOLMES is superior in DNA detection, and SHERLOCK is more suitable in RNA detection. In addition to biological samples, HOLMES can be used to detect pathogen-contaminated food and environment [64].

#### 3.3.4. Other CRISPR/Cas-Based Detection Methods

Traditional detection methods such as DFA and RT–PCR fail to detect the virus at the early stage of rabies virus (RABV) infection. Ren et al. [56] developed the RPA–CRISPR method and found that this method can significantly improve the sensitivity of viral detection, and detect viral RNA in cerebrospinal fluid samples at 3 days postinfection. Hence, the RPACRISPR method enables early diagnosis of RABV, thereby possessing great application potential in the early diagnosis of human RABV infection [56]. Due to its high sensitivity, RPA–CRISPR can be used not only for the detection of pathogenic microorganisms such as viruses, parasites, and bacteria under laboratory conditions, but also for field detection, single nucleotide polymorphism detection, miRNA quantification, and species identification in ecological studies [25, 65–67]. Therefore, RPACRISPR method will have broader development and application prospects. CRISPR/Cas12a has been shown to be a sensitive and specific method for detecting human papillomavirus (HPV) DNA in anal swabs. However, the existing CRISPR/Cas12a system requires expensive and auxiliary equipment, limiting the application as a point of care (POC) diagnostic instrument. To address such limitation, Tsou et al. [68] aimed to develop a CRISPR/Cas12a-based POC test that directly targets plasma for circulating HPV DNA detection and can be read immediately with naked eyes. In their research, they used cell-cultured supernatants of HPV16- or 18-positive cancer cells, treated them with lysis buffer, and performed isothermal amplification without DNA isolation. They incubated Cas12a, crRNA, and fluorescent–biotin reporters with the lysates, and integrated the CRISPR/Cas12a with lateral-flow strips for specific and direct detection of HPV16 and 18 in the liquid samples. The test demonstrated a limit of detection (0.24 femtomolar), which was comparable to polymerase chain reaction but required less time. Yu et al. [55] established a rapid and accurate detection method for *Cryptosporidium parvum* based on thermostatic amplification technology and Cas12a/crRNA trans-cleavage system (ReCTC). Fluorescence and lateral flow test strip analysis of ReCTC does not require specialized auxiliary equipment or power supply. The sensitivity can reach 1 and 10 copies/*μ*L, respectively, in the detection of pure and complex samples, with high specificity. Notably, no cross-reactivity was observed between *C. parvum* and *Cryptococcus microplus*, or other common intestinal parasite protozoan subtypes when detected by this method.

CRISPR/Cas system has advantages in pathogen detection due to its high sensitivity, simplicity of operation, and ability to achieve rapid and immediate detection. Traditional nucleic acid detection techniques such as PCR are time-consuming and require sophisticated instruments and professional operators. On the contrary, CRISPR/Cas-based detection methods such as SHERLOCK and HOLMES combined with RPA can achieve rapid, highly sensitive, and specific field detection, thereby making an important contribution to the early and rapid diagnosis of pathogenic microorganisms. Moreover, with the cost reduction and further technique promotion, CRISPR/Cas-based methods are expected to be more widely used in the detection of zoonosis in the future.

### 3.4. Vaccine Development-Based CRISPR/Cas

In addition to pathogenesis research and detection of pathogenic microorganisms, CRISPR/Cas system is widely used in vaccine research, providing an efficient and powerful strategy for the development of genetically engineered vaccines.

Molecular epidemiological studies have shown that pseudorabies virus has undergone significant genetic variation and that there exist a large number of alterations such as deletions, insertions, and substitutions in pseudorabies viral variants. The currently commercially available vaccine Bartha-K61 provides partial protection against prevalent pseudorabies virus variants such as HeN1 and TJ [69–71]. CRISPR/Cas system can be utilized to efficiently edit and modify existing vaccine strains to match the antigenic variation of currently prevalent strains and to improve the reproduction efficiency of vaccine strains. For example, Yu et al. [4] employed CRISPR/Cas9 system to directly perform large segment replacement editing on viral genes. In the experiments, *gB* gene of the classic PRV vaccine strain Bartha-K61 was replaced with that of the PRV mutant strain JS-2012 by employing CRISPR/Cas9 system combined with homologous recombination to generate the recombinant virus rPRV-BJB [4]. In another example, Zhao et al. [35] constructed PR triple gene-defective strains using CRISPR/Cas9 system, and the modified trains showed high efficacy and safety in challenge experiments. Similarly, Tang et al. [72] generated a triple gene-defective strain HeN1 PRV by editing the PRV genome based on CRISPR/Cas9 system. Further assessment of PRV attenuation in mice revealed that the modified PRV strain was significantly less virulent [9]. All these studies demonstrate that CRISPR/Cas9 system enables a rapid antigenic matching of vaccine strains to prevalent strains as well as attenuation of the virulence of the strains, thus allowing for rapid adaptation of vaccine strain change to the variation of prevalent strains to achieve good immunity.

Herpesvirus of Turkeys (HVT) functions as a vector platform for developing recombinant vaccines against a range of avian diseases, including infectious bursal disease (IBD). It also serves as a live vaccine against Marek's disease (MD) [73, 74]. Tang et al. [22] developed a method for constructing HVT recombinants for efficient and rapid expression of IBDV VP2 protein by combining a vector with excisable red fluorescent protein (RFP) flanked by LoxP sites and unique restriction endonuclease sites with sgRNA. This method provides an effective means to incorporate additional viral antigens into the HVT genome, facilitating the rapid development of recombinant vaccines against a variety of avian diseases, such as infectious laryngotracheitis, Avian influenza, Newcastle disease, avian leukosis, and coccidiosis [75–77]. Among these, infectious laryngotracheitis virus (ILTV) stands out as a promising vaccine vector due to its ability to accommodate heterologous gene, low pathogenicity, and potential to induce cellular and humoral arms of immunity. However, using the conventional recombination methods, the gene editing process is time-demanding, and error prone. Compared with traditional methods, CRISPR/Cas showed its advantages. For example, Atasoy et al. [78] used CRISPR/Cas9 and Cre–Lox systems to delete the thymidine kinase and unique short four gene of ILTV, and insert the fusion gene of Newcastle disease virus to develop a vaccine candidate capable of preventing infectious ILTV and NDV. Assessment of the stable expression, replication kinetics, and in vitro properties of the inserted protein of this potential vaccine candidate indicates that CRISPR/Cas9 combined with Cre–Lox system provides an operative method for rapid development of ILTV-based recombinant vaccines. This method can not only delete the virulence factor of ILTV but also insert heterologous genes to develop polyvalent vaccine strains. CRISPR/Cas9 system can also significantly increase the efficiency of developing recombinant ASFV. Based on the CRISPR/Cas9 system, by using porcine macrophages as the cell substrate, Borca et al. [79] successfully constructed a recombinant virus by deleting the nonessential gene 8-DR from the genome of the highly virulent wild strain Georgia 07 and replacing it with RFP gene. Using CRISPR/Cas9 system, Abkallo et al. [80] achieved the development of a recombinant virus by eliminating the nonessential gene *A238L* from the genome of the highly virulent genotype IX ASFV (ASFV-Kenya-IX-1033). This accomplishment was accomplished in a significantly shorter timeframe of less than 2 months, in contrast to the conventional homologous recombination methods combined with conventional purification techniques, which typically require an average of 6 months, indicating that CRISPR/Cas technique can accelerate vaccine development for ASF [80]. Thus, CRISPR/Cas-based editing of ASFV genome is a crucial instrument for developing ASF vaccines.

CRISPR/Cas9 system is also a valuable tool for the research and development of bacterial vaccines. Wang et al. [81] generated a double gene deletion strain *Δ*SopBSsrB as well as two single deletion strains *Δ*SopB and *Δ*SsrB using CRISPR/Cas9 system. Further studies showed that all the three *Salmonella typhimurium* gene deletion strains had attenuated virulence and reduced pathogenicity in mice [82]. In another study, Yu et al. [83] knocked out the virulence-related *aroA* gene of *E. coli* using CRISPR/Cas9 system, laying a foundation for the development of attenuated *E. coli* vaccines.

CRISPR/Cas9 system also had applicability to knock out the virulence gene of parasites for the construction of vaccine strains. For example, In the study conducted by Yang et al. [84] the CRISPR/Cas9 system was utilized to knock out the *NPT1* gene in the Type I *T*. *gondii* strain, resulting in the creation of the RH:*Δ*NPT1 strain. The researchers studied the potential of this strain as a live attenuated vaccine against *Toxoplasma gondii* infection [84]. The study demonstrated that vaccination with the RH:*Δ*NPT1 strain induced robust cellular and humoral immune responses in mice. Importantly, it was found that the vaccinated mice were effectively protected against *T. gondii* infection. Moreover, RH:*Δ*NPT1 can not only prevent acute infection of various genotypes of *T. gondii*, but also improve survival and alleviate the response of chronic infections such as cerebral cysts [6]. In other studies, researchers successfully carried out gene knockout on a number of genes in *T. gondii*, such as *granule protein 17* (*gra17*), *novel putative transporter* (*npt1*), *tkl1*, and *cdpk2*, using CRISPR/Cas9 [85]. These studies show that CRISPR/Cas system can be used to rapidly edit genes in *T. gondii* for the construction of attenuated vaccine strains.

### 3.5. Typing of Pathogenic Microorganisms Using CRISPR/Cas

CRISPR/Cas is the fastest-evolving genetic element in bacterial genomes and is distributed in about 40% of bacteria and 90% of archaea [86]. Based on the differences of CRISPR spacers, the corresponding primers can be designed to amplify CRISPR in bacteria and obtain CRISPR sequence information. Further alignment and analysis of the CRISPR sequence information allows for bacterial typing [87]. Fabre et al. [88] performed CRISPR analysis on 783 *Salmonella* strains of 130 serotypes and found that compared with pulsed field gel electrophoresis (PFGE), CRISPR can more effectively distinguish strains of the same serotype in outbreaks. Subsequent studies showed that CRISPR is less time-consuming and more automated, while it can be used to study the typing of bacteria, such as *Streptococcus agalactiae*, *Staphylococcus lugdunensis*, and *Cronobacter spp*. [89]. In addition to being used alone in the typing of *Salmonella*, CRISPR can be used in combination with other genotyping methods. Liu et al. [59] developed a new typing method called CRISPR–MVLST by combining multilocus sequence typing (MLST) with CRISPR, and performed typing on 171 clinical *Salmonella* isolates of nine serotypes using CRISPR-MVLST. Compared with PFGE, CRISPR-MVLST displays higher resolution and can distinguish outbreak strains from highly cloned strains [90]. Therefore, CRISPR/Cas technique can provide us high-resolution tools for serological or genetic typing.

### 3.6. CRISPR/Cas Techniques for Other Areas

The generation of disease models plays a crucial role in developing new therapeutic strategies and comprehending disease mechanisms. The utilization of CRISPR/Cas has become widespread in the establishment of disease-related cellular models, encompassing various conditions such as duchenne muscular dystrophy (DMD) [29], aniridia-related keratopathy [65], brittle bone [91], X-linked adrenoleukodystrophy [92], and Alzheimer's disease [74]. In addition, researchers have established a series of mouse models employing CRISPR/Cas that recapitulate DMD [73], atherosclerosis [75], obesity and diabetes [93], RTH*α* [94], and Alzheimer's disease [95]. Moreover, CRISPR/Cas has been employed to develop disease models in large animals, including sheep [87], rabbit [26], pig [96], and monkey [97]. CRISPR/Cas technology provides a user-friendly and highly adaptable means for development of disease models, allowing researchers to delve into the genetic underpinnings of diseases and assess potential therapeutic strategies.

Monogenic diseases have a significant impact on a large population of patients. The ClinVar database has identified over 75,000 pathogenic genetic variants associated with these diseases [5, 98]. Clinical trials using CRISPR have already been initiated for several inherited diseases, including Leber congenital amaurosis LCA10, *β*-thalassemia, sickle cell disease, and transfusion-dependent *β*-thalassemia [64]. These clinical trials mark the beginning of CRISPR/Cas-based gene therapy, offering promising prospects for treating genetic diseases, especially those currently considered incurable, and enhancing cell-based therapies.

## 4. Challenge and Prospective

With the rapid development of urbanization and modern animal husbandry, new emerging and reemerging zoonosis was spreading world widely. Zoonotic diseases have profound consequences on both animal and human health, as well as on economies and livelihoods. Zoonotic pathogens, such as influenza and SARS, account for most emerging infectious diseases in people [82]. It is noteworthy that over three-quarters of these emerging zoonotic pathogens have their origins in wildlife [99]. Therefore, it is of great significance to develop rapid diagnostic methods, vaccines, and drugs, and breed disease-resistant animals. New tools are needed for the above-mentioned researches. Based on CRISPR/Cas system, new platforms of pathogen detection and genetic editing were established, significantly advancing our understanding of microbe-host interactions. These platforms also provided practical applications in the development of novel diagnostics and vaccines for zoonosis. Besides, CRISPR/Cas system can also be used for the development of antiviral drugs. However, there are few studies on drugs against zoonosis by using CRISPR/Cas system. Furthermore, viral pathogens such as influenza virus and coronavirus have numerous serotypes, so universal vaccines are useful tools for antivirus. However, the genetic tools or methods were limited for the development of universal vaccines.

In order to take advantages of this technique, we not only need to understand the biology of CRISPR and superiorities, but also need to know the shortcomings of this technique. Like other gene editing methods, CRISPR/Cas system also has its limitations. For example, in some bacteria, CRISPR/Cas9 shows toxicity to cells and is sensitive to the level of intracellular Cas9 [26]. In other circumstances, once the sgRNA recognizes a nonspecific site rather than the target sequence in the genome and mediates the cleavage, an “off-target” effect will occur [96]. This effect could limit the application of CRISPR technique. In fact, a large number of studies on the modification of Cas9 protein have been initiated to expand the targeting range of CRISPR/Cas9 system and to improve targeting specificity by solving the off-target problems [100, 101]. Moreover, when DNA-targeting CRISPR/Cas system is used to counteract viruses, it needs to be considered that escape mutations can generate Cas9-resistant variants, which could cause the generation of more pathogenic mutant viruses [89]. For the sake of better using CRISPR/Cas system for the research and application of zoonosis, this technique requires further improvement. For example, in terms of vaccine development, viral vector vaccines carrying CRISPR/Cas9 components may potentially cause adverse effects due to the off-target effects. Thus, risk reduction is one of the main directions in the development of this type of vaccine. In addition, the delivery media for CRISPR/Cas9 plasmids need further development, and the safety and efficacy of various delivery media require further assessment. In order to establish a more convenient field-detection method, the CRISPR/Cas system should be applied, under the condition of minimizing the steps of nucleic acid extraction and amplification, and without the expense of the detection sensitivity.

For overcoming the shortcomings, we need to use chemical and biotechnical tools and methods. For example, using chemical modifications in specific positions of sgRNAs could improve binding affinity. Likewise, phosphate backbone modifications enhance nuclease resistance and resistance [16]. The delivery of CRISPR/Cas components poses a significant challenge for the widespread application of CRISPR/Cas. To overcome this problem, researchers have focused on developing smaller variants of the Cas9, which are derived from *Staphylococcus aureus*, known as SaCas9 [90], and *Neisseria meningitides*, known as Nme2Cas9 [102]. These smaller Cas9 proteins have demonstrated gene editing efficiency comparable to that of SpCas9. Importantly, their smaller size makes them more amenable for in vivo delivery compared to the larger SpCas9 (∼4.3 kb). Moreover, delivery systems such as nanoparticles should be developed and tested. For example, a recent study showed that PEG–PEI nanoparticles have low cytotoxicity and can effectively deliver mRNAs to immune cells in the lung and play regulatory roles [20]. However, it is unclear whether this system is suitable for the delivering CRISPR components and further investigation is warranted.

This study reviews the principles and applications of CRISPR/Cas system in zoonosis, including researching on the pathogenesis, rapid diagnosis, vaccine development, editing of disease-resistant genes, typing of pathogens and, etc. With the continuous development and improvement of the CRISPR/Cas system, this technique will be widely used in the studies of the transcriptome and proteome of animals, viruses, bacteria, parasites, etc., which will lead to a better understanding of the genome function, expression, and regulatory mechanism, thereby laying a basis for effective prevention and treatment of zoonosis. Alternatively, the development and application of CRISPR/Cas system in control and prevention of zoonosis can also be extended in other areas, such as, development of antiviral drugs. Therefore, the CRISPR/Cas tool will be a more powerful weapon to battle with zoonosis.

## Figures and Tables

**Table 1:** Classification of endonucleases commonly used in gene editing.

Endonuclease type	Constitute	Advantage	Disadvantage	Reference
MNs	Endonuclease of DNA	Specific cleavage of long sequences on the DNA strand	Low cleavage efficiency	[7]

ZFNs	Artificial fusion proteins, including the DNA recognition domain and the endonuclease FokI	Applying in many species and genetic loci	Operational complexity, time-consuming, and off-target effects	[8]

TALENs	TALE protein, FokI nuclease	Targeting at specific genes in different species	Off-target effects, the specific binding is easily affected	[11]

CRISPR/Cas	Cas9 endonuclease, crRNA, and tracrRNA	Simultaneously editing multiple genes, accurate targeting, low off-target rate, low cytotoxicity, cheaper, and simpler	sgRNA design tools directly affect editing efficiency and targeting specificity	[14]

**Table 2:** Brief summary of CRISPR/Cas systems used in gene editing.

Class	Type	Subtypes	Signature genes	Effector proteins	Target
Class 1	Type Ⅰ	I-A∼I-F, I-U	Cas3	Multisubunit complex (signature protein Cas3)	dsDNA
	Type Ⅲ	III-A∼Ⅲ-F	Cas10	Multisubunit complex (signature protein Cas10)	mRNA/DNA
	Type Ⅳ	IV-A, IV-B	Csf1	Multisubunit complex (signature protein Csf1)	Unknown

Class 2	Type Ⅱ	II-A∼II-C	Cas9	Cas9	dsDNA
	Type Ⅴ	Ⅴ-A∼Ⅴ-I, V-U	Cpf1	Cas12a	dsDNA/ssDNA
			C2c1	Cas12b	dsDNA/ssDNA
	Type Ⅵ		C2c2	Cas13	ssRNA

ssDNA, single-stranded DNA; dsDNA, double-stranded DNA.

**Table 3:** Summary of CRISPR/Cas-based diagnostic tools.

Method	Protein	Target	Amplification	Detection	Reference
DETECTR	Cas12a	DNA	RPA	Fluorescence	[53]
HOLMES	Cas12a	DNA, RNA	PCR	Fluorescence	[14]
SHERLOCK	Cas13a	DNA, RNA	RPA	Fluorescence	[52]
SHERLOCKv2	Cas13a	RNA	RPA	Fluorescence/colorimetry	[54]
ReCTC	Cas12a	DNA	RPA	Fluorescence	[55]
RPA–CRISPR	Cas12a	DNA, RNA	RPA	Fluorescence	[56]

## Data Availability

Data sharing not applicable to this article as no datasets were generated during the current study.

## References

[1] Shrivastava S. R., Shrivastava P. S., Ramasamy J. (2015). Neglected zoonotic diseases: it’s now time for action urges WHO. *Journal of Research in Medical Sciences*.

[2] Vajda Á., Ózsvári L., Szakos D., Kasza G. (2021). Estimation of the Impact of foodborne salmonellosis on consumer well-being in hungary. *International Journal of Environmental Research and Public Health*.

[3] Li H., Yang Y., Hong W., Huang M., Wu M., Zhao X. (2020). Applications of genome editing technology in the targeted therapy of human diseases: mechanisms, advances and prospects. *Signal Transduction and Targeted Therapy*.

[4] Yu Z.-Q., Tong W., Zheng H. (2017). Variations in glycoprotein B contribute to immunogenic difference between PRV variant JS-2012 and Bartha-K61. *Veterinary Microbiology*.

[5] Anzalone A. V., Randolph P. B., Davis J. R. (2019). Search-and-replace genome editing without double-strand breaks or donor DNA. *Nature*.

[6] Boissel S., Jarjour J., Astrakhan A. (2014). megaTALs: a rare-cleaving nuclease architecture for therapeutic genome engineering. *Nucleic Acids Research*.

[7] Lambert A. R., Hallinan J. P., Shen B. W. (2016). Indirect DNA Sequence recognition and its impact on nuclease cleavage activity. *Structure*.

[8] Porter S. N., Levine R. M., Pruett-Miller S. M. (2019). A practical guide to genome editing using targeted nuclease technologies. *Comprehensive Physiology*.

[9] Stoddard B. L. (2014). Homing endonucleases from mobile group I introns: discovery to genome engineering. *Mobile DNA*.

[10] Paschon D. E., Lussier S., Wangzor T. (2019). Diversifying the structure of zinc finger nucleases for high-precision genome editing. *Nature Communications*.

[11] Mussolino C., Cathomen T. (2012). TALE nucleases: tailored genome engineering made easy. *Current Opinion in Biotechnology*.

[12] Kaneko T., Hatada I. (2023). Genome editing of rat. *Genome Editing in Animals*.

[13] Yum S.-Y., Choi W., Kim S., Jang G., Koo O. (2023). Identification *AAVS1*-like locus from the porcine genome and site-specific integration of recombinase-mediated cassette exchange using CRISPR/Cas9. *Animal Biotechnology*.

[14] Li S.-Y., Cheng Q.-X., Wang J.-M. (2018). CRISPR-Cas12a-assisted nucleic acid detection. *Cell Discovery*.

[15] Chen L., Tang L., Xiang H. (2014). Advances in genome editing technology and its promising application in evolutionary and ecological studies. *GigaScience*.

[16] Castro N. G., Bjelic J., Malhotra G., Huang C., Alsaffar S. H. (2021). Comparison of the feasibility, efficiency, and safety of genome editing technologies. *International Journal of Molecular Sciences*.

[17] Maeder M. L., Stefanidakis M., Wilson C. J. (2019). Development of a gene-editing approach to restore vision loss in leber congenital amaurosis Type 10. *Nature Medicine*.

[18] Tyler McCullough K., Boye S. L., Fajardo D. (2019). Somatic gene editing of *GUCY2D* by AAV-CRISPR/Cas9 alters retinal structure and function in mouse and macaque. *Human Gene Therapy*.

[19] Firth A L., Menon T., Parker G S. (2015). Functional gene correction for cystic fibrosis in lung epithelial cells generated from patient iPSCs. *Cell Reports*.

[20] Ke X., Shelton L., Hu Y. (2020). Surface-functionalized PEGylated nanoparticles deliver messenger RNA to pulmonary immune cells. *ACS Applied Materials & Interfaces*.

[21] Tian S., Jiang L., Gao Q. (2017). Efficient CRISPR/Cas9-based gene knockout in watermelon. *Plant Cell Reports*.

[22] Tang N., Zhang Y., Pedrera M. (2018). A simple and rapid approach to develop recombinant avian herpesvirus vectored vaccines using CRISPR/Cas9 system. *Vaccine*.

[23] Char S. N., Neelakandan A. K., Nahampun H. (2017). An *Agrobacterium*-delivered CRISPR/Cas9 system for high-frequency targeted mutagenesis in maize. *Plant Biotechnology Journal*.

[24] Liu X., Wu S., Xu J., Sui C., Wei J. (2017). Application of CRISPR/Cas9 in plant biology. *Acta Pharmaceutica Sinica B*.

[25] Xu J.-H., Kang L., Yuan B. (2022). Development and evaluation of a rapid RPA/CRISPR-based detection of *Francisella tularensis*. *Frontiers in Microbiology*.

[26] Xu Y., Wang Y., Song Y. (2018). Generation and phenotype Identification of *PAX4* gene knockout rabbit by CRISPR/Cas9 system. *G3 Genes|Genomes|Genetics*.

[27] Snijder E. J., Kikkert M., Fang Y. (2013). Arterivirus molecular biology and pathogenesis. *Journal of General Virology*.

[28] Na L., Shuqi X., Chong Z., Anke Z., Gaopeng H., Enmin Z. (2014). Gene editing and functional study of PRRSV receptor NMHC II-A using TALEN and CRISPR-Cas9 system, (in Chinese), China society of animal husbandry and veterinary medicine, animal husbandry and veterinary biotechnology branch and china society of immunology, veterinary immunology branch symposium.

[29] Shimo T., Hosoki K., Nakatsuji Y., Yokota T., Obika S. (2018). A novel human muscle cell model of Duchenne muscular dystrophy created by CRISPR/Cas9 and evaluation of antisense-mediated exon skipping. *Journal of Human Genetics*.

[30] Wen X., Ge X., Zhou L., Zhang Y., Guo X., Yang H. (2021). PRRSV promotes MARC-145 cells entry into S phase of the cell cycle to facilitate viral replication *via* degradation of p21 by nsp11. *Frontiers in Veterinary Science*.

[31] Li W., Luo R., He Q., van Kuppeveld F. J. M., Rottier P. J. M., Bosch B.-J. (2017). Aminopeptidase N is not required for porcine epidemic diarrhea virus cell entry. *Virus Research*.

[32] Liu Z., Zhang M., Huang P. (2023). Generation of APN-chimeric gene-edited pigs by CRISPR/Cas9-mediated knock-in strategy. *Gene*.

[33] Chen W., Zhang Y., Zhang Y. (2018). CRISPR/Cas9-based genome editing in *Pseudomonas aeruginosa* and cytidine deaminase-mediated base editing in *Pseudomonas* species. *iScience*.

[34] Synefiaridou D., Veening J.-W. (2021). Harnessing CRISPR-Cas9 for genome editing in *Streptococcus pneumoniae* D39V. *Applied and Environmental Microbiology*.

[35] Zhao Y., Wang L.-Q., Zheng H.-H. (2020). Construction and immunogenicity of a gE/gI/TK-deleted PRV based on porcine pseudorabies virus variant. *Molecular and Cellular Probes*.

[36] Hong W., Zhang J., Cui G., Wang L., Wang Y. (2018). Multiplexed CRISPR-Cpf1-mediated genome editing in clostridium difficile toward the understanding of pathogenesis of *C. difficile* infection. *ACS Synthetic Biology*.

[37] Vigouroux A., Oldewurtel E., Cui L., Bikard D., van Teeffelen S. (2018). Tuning dCas9’s ability to block transcription enables robust, noiseless knockdown of bacterial genes. *Molecular Systems Biology*.

[38] Choudhary E., Thakur P., Pareek M., Agarwal N. (2015). Gene silencing by CRISPR interference in mycobacteria. *Nature Communications*.

[39] Hatoum-Aslan A., Marraffini L. A. (2014). Impact of CRISPR immunity on the emergence and virulence of bacterial pathogens. *Current Opinion in Microbiology*.

[40] Huang J., Sun B., Zhang X. (2010). *Shewanella xiamenensis* sp. nov., isolated from coastal sea sediment. *International Journal of Systematic and Evolutionary Microbiology*.

[41] Wang J.-H., Tseng S.-Y., Tung K.-C. (2020). Genomic investigation of emerging zoonotic pathogen *Shewanella xiamenensis*. *Tzu Chi Medical Journal*.

[42] Cathomen T., Keith Joung J. (2008). Zinc-finger nucleases: the next generation emerges. *Molecular Therapy*.

[43] Fernandes L. G. V., Guaman L. P., Vasconcellos S. A., Heinemann M. B., Picardeau M., Nascimento A. L. T. O. (2019). Gene silencing based on RNA-guided catalytically inactive Cas9 (dCas9): a new tool for genetic engineering in *Leptospira*. *Scientific Reports*.

[44] Young J., Dominicus C., Wagener J. (2019). A CRISPR platform for targeted in vivo screens identifies *Toxoplasma gondii* virulence factors in mice. *Nature Communications*.

[45] Luo L., Wang S., Zhu L. (2019). Aminopeptidase N-null neonatal piglets are protected from transmissible gastroenteritis virus but not porcine epidemic diarrhea virus. *Scientific Reports*.

[46] Chin K.-C., Cresswell P. (2001). Viperin (cig5), an IFN-inducible antiviral protein directly induced by human cytomegalovirus. *Proceedings of the National Academy of Sciences of the United States of America*.

[47] Li W., Mao L., Cao Y. (2017). Porcine viperin protein inhibits the replication of classical swine fever virus (CSFV) in vitro. *Virology Journal*.

[48] Nasr N., Maddocks S., Turville S. G. (2012). HIV-1 infection of human macrophages directly induces *viperin* which inhibits viral production. *Blood*.

[49] Xie Z., Jiao H., Xiao H. (2020). Generation of p*RSAD2* gene knock-in pig via CRISPR/Cas9 technology. *Antiviral Research*.

[50] Whitworth K. M., Lee K., Benne J. A. (2014). Use of the CRISPR/Cas9 system to produce genetically engineered pigs from in vitro-derived oocytes and embryos. *Biology of Reproduction*.

[51] Whitworth K. M., Rowland R. R. R., Ewen C. L. (2016). Gene-edited pigs are protected from porcine reproductive and respiratory syndrome virus. *Nature Biotechnology*.

[52] Gootenberg J. S., Abudayyeh O. O., Lee J. W. (2017). Nucleic acid detection with CRISPR-Cas13a/C2c2. *Science*.

[53] Chen J. S., Ma E., Harrington L. B. (2018). CRISPR-Cas12a target binding unleashes indiscriminate single-stranded DNase activity. *Science*.

[54] Gootenberg J. S., Abudayyeh O. O., Kellner M. J., Joung J., Collins J. J., Zhang F. (2018). Multiplexed and portable nucleic acid detection platform with Cas13, Cas12a, and Csm6. *Science*.

[55] Yu F., Zhang K., Wang Y. (2021). CRISPR/Cas12a-based on-site diagnostics of *Cryptosporidium parvum* IId-subtype-family from human and cattle fecal samples. *Parasites & Vectors*.

[56] Ren M., Mei H., Zhou J., Zhou M., Han H., Zhao L. (2021). Early diagnosis of rabies virus infection by RPA-CRISPR techniques in a rat model. *Archives of Virology*.

[57] Li Z., Wei J., Di D. (2020). Rapid and accurate detection of African swine fever virus by DNA endonuclease-targeted CRISPR trans reporter assay. *Acta Biochimica et Biophysica Sinica*.

[58] Broughton J. P., Deng X., Yu G. (2020). CRISPR-Cas12-based detection of SARS-CoV-2. *Nature Biotechnology*.

[59] Liu F., Barrangou R., Gerner-Smidt P., Ribot E. M., Knabel S. J., Dudley E. G. (2011). Novel virulence gene and clustered regularly interspaced short palindromic repeat (CRISPR) multilocus sequence typing scheme for subtyping of the major serovars of *Salmonella enterica* subsp. *enterica*. *Applied and Environmental Microbiology*.

[60] Lee R. A., De Puig H., Nguyen P. Q. (2020). Ultrasensitive CRISPR-based diagnostic for field-applicable detection of *Plasmodium* species in symptomatic and asymptomatic malaria. *Proceedings of the National Academy of Sciences of the United States of America*.

[61] Patchsung M., Jantarug K., Pattama A. (2020). Clinical validation of a Cas13-based assay for the detection of SARS-CoV-2 RNA. *Nature Biomedical Engineering*.

[62] Joung J., Ladha A., Saito M. (2020). Detection of SARS-CoV-2 with SHERLOCK one-pot testing. *New England Journal of Medicine*.

[63] Aman R., Ali Z., Butt H. (2018). RNA virus interference via CRISPR/Cas13a system in plants. *Genome Biology*.

[64] Wu S.-S., Li Q.-C., Yin C.-Q., Xue W., Song C.-Q. (2020). Advances in CRISPR/Cas-based gene therapy in human genetic diseases. *Theranostics*.

[65] Roux L. N., Petit I., Domart R. (2018). Modeling of aniridia-related keratopathy by CRISPR/Cas9 genome editing of human limbal epithelial cells and rescue by recombinant PAX6 protein. *Stem Cells*.

[66] Pankowicz F. P., Barzi M., Legras X. (2016). Reprogramming metabolic pathways *in vivo* with CRISPR/Cas9 genome editing to treat hereditary tyrosinaemia. *Nature Communications*.

[67] Kemaladewi D. U., Bassi P. S., Erwood S. (2019). A mutation-independent approach for muscular dystrophy via upregulation of a modifier gene. *Nature*.

[68] Tsou J.-H., Leng Q., Jiang F. (2019). A CRISPR test for detection of circulating nuclei acids. *Translational Oncology*.

[69] An T.-Q., Peng J.-M., Tian Z.-J. (2013). Pseudorabies virus variant in Bartha-K61–vaccinated pigs, China, 2012. *Emerging Infectious Diseases*.

[70] Zhou M., Wu X., Jiang D. (2019). Characterization of a moderately pathogenic pseudorabies virus variant isolated in China, 2014. *Infection, Genetics and Evolution*.

[71] Luo Y., Li N., Cong X. (2014). Pathogenicity and genomic characterization of a pseudorabies virus variant isolated from Bartha-K61–vaccinated swine population in China. *Veterinary Microbiology*.

[72] Tang Y.-D., Liu J.-T., Wang T.-Y. (2016). Live attenuated pseudorabies virus developed using the CRISPR/Cas9 system. *Virus Research*.

[73] Egorova T. V., Zotova E. D., Reshetov D. A. (2019). CRISPR/Cas9-generated mouse model of Duchenne muscular dystrophy recapitulating a newly identified large 430 kb deletion in the human *DMD* gene. *Disease Models & Mechanisms*.

[74] Paquet D., Kwart D., Chen A. (2016). Efficient introduction of specific homozygous and heterozygous mutations using CRISPR/Cas9. *Nature*.

[75] Jarrett K. E., Lee C., De Giorgi M. (2018). Somatic editing of *Ldlr* with adeno-associated viral-CRISPR is an efficient tool for atherosclerosis research. *Arteriosclerosis, Thrombosis, and Vascular Biology*.

[76] Bjursell M., Porritt M. J., Ericson E. (2018). Therapeutic genome editing with CRISPR/Cas9 in a humanized mouse model ameliorates *α*1-antitrypsin deficiency phenotype. *EBioMedicine*.

[77] Alapati D., Zacharias W. J., Hartman H. A. (2019). In utero gene editing for monogenic lung disease. *Science Translational Medicine*.

[78] Atasoy M. O., Rohaim M. A., Munir M. (2019). Simultaneous deletion of virulence factors and insertion of antigens into the infectious laryngotracheitis virus using NHEJ-CRISPR/Cas9 and Cre–Lox system for construction of a stable vaccine vector. *Vaccines*.

[79] Borca M. V., Holinka L. G., Berggren K. A., Gladue D. P. (2018). CRISPR-Cas9, a tool to efficiently increase the development of recombinant African swine fever viruses. *Scientific Reports*.

[80] Abkallo H. M., Hemmink J. D., Oduor B. (2022). Co-deletion of A238L and EP402R genes from a genotype IX African swine fever virus results in partial attenuation and protection in swine. *Viruses*.

[81] Wang Q.-Y. (2021). *Construction and Pathogenicity Analysis of Multiple Gene Deletion Strains of Salmonella Typhimurium, (In Chinese)*.

[82] Taylor L. H., Latham S. M., Woolhouse M. E. J. (2001). Risk factors for human disease emergence. *Philosophical Transactions of the Royal Society of London. Series B: Biological Sciences*.

[83] Yu Z., Jinrong Z., Linghong Z., Erpeng Z., Wudou, Baocheng W. (2016). Construction of a knockout system for the aroA gene in *Escherichia coli* using CRISPR/Cas9 technology and its preliminary application (in Chinese). *Journal of Animal Husbandry and Veterinary Medicine*.

[84] Yang W.-B., Wang J.-L., Gui Q. (2019). Immunization with a live-attenuated RH: *ΔNPT1* strain of *Toxoplasma gondii* induces strong protective immunity against toxoplasmosis in mice. *Frontiers in Microbiology*.

[85] Wang J.-L., Li T.-T., Elsheikha H. M. (2018). Live attenuated Pru: *Δcdpk2* strain of *Toxoplasma gondii* protects against acute, chronic, and congenital toxoplasmosis. *The Journal of Infectious Diseases*.

[86] Horvath P., Barrangou R. (2010). CRISPR/Cas, the immune system of bacteria and archaea. *Science*.

[87] Fan Z., Perisse I. V., Cotton C. U. (2018). A sheep model of cystic fibrosis generated by CRISPR/Cas9 disruption of the *CFTR* gene. *JCI Insight*.

[88] Fabre L., Zhang J., Guigon G. (2012). CRISPR typing and subtyping for improved laboratory surveillance of *Salmonella* infections. *PLOS ONE*.

[89] Shen C., Li X., Wu Y. (2023). Application of CRISPR/Cas gene editing system in plant antiviral defense. (In Chinese). *Journal of Phytopathology*.

[90] Ann Ran F., Cong L., Yan W. X. (2015). *In vivo* genome editing using *Staphylococcus aureus* Cas9. *Nature*.

[91] Far H. H., Patria Y. N., Motazedian A. (2019). Generation of a heterozygous *COL1A1* (c.3969_3970insT) osteogenesis imperfecta mutation human iPSC line, MCRIi001-A-1, using CRISPR/Cas9 editing. *Stem Cell Research*.

[92] Raas Q., Gondcaille C., Hamon Y. (2019). CRISPR/Cas9-mediated knockout of *Abcd1* and *Abcd2* genes in BV-2 cells: novel microglial models for X-linked adrenoleukodystrophy. *Biochimica et Biophysica Acta (BBA) - Molecular and Cell Biology of Lipids*.

[93] Roh J.-I., Lee J., Park S. U. (2018). CRISPR-Cas9-mediated generation of obese and diabetic mouse models. *Experimental Animals*.

[94] Markossian S., Guyot R., Richard S. (2018). CRISPR/Cas9 editing of the mouse *Thra* gene produces models with variable resistance to thyroid hormone. *Thyroid*.

[95] Tan D. C. S., Yao S., Ittner A. (2018). Generation of a new tau knockout (tau^*Δ*ex1^) line using CRISPR/Cas9 genome editing in mice. *Journal of Alzheimer’s Disease*.

[96] Yan S., Tu Z., Liu Z. (2018). A huntingtin knockin pig model recapitulates features of selective neurodegeneration in huntington’s disease. *Cell*.

[97] Yang W., Li S., Li X.-J. (2019). A CRISPR monkey model unravels a unique function of PINK1 in primate brains. *Molecular Neurodegeneration*.

[98] Landrum M. J., Lee J. M., Benson M. (2016). ClinVar: public archive of interpretations of clinically relevant variants. *Nucleic acids research*.

[99] Jones K. E., Patel N. G., Levy M. A. (2008). Global trends in emerging infectious diseases. *Nature*.

[100] Naeem M., Alkhnbashi O. S. (2023). Current bioinformatics tools to optimize CRISPR/Cas9 experiments to reduce off-target effects. *International Journal of Molecular Sciences*.

[101] Lee J., Lim K., Kim A. (2023). Prime editing with genuine cas9 nickases minimizes unwanted indels. *Nature Communications*.

[102] Edraki A., Mir A., Ibraheim R. (2019). A compact, high-accuracy cas9 with a dinucleotide PAM for *in vivo* genome editing. *Molecular Cell*.

